# Using Google Trends to assess the impact of global public health days on online health information seeking behaviour in Central and South America

**DOI:** 10.7189/jogh.10.010403

**Published:** 2020-06

**Authors:** Eva Maria Havelka, Christian David Mallen, Thomas Andrew Shepherd

**Affiliations:** 1 School of Medicine, Keele University, Staffordshire, UK; 2 School of Primary, Community and Social Care, Keele University, Staffordshire, UK

## Abstract

**Background:**

Public health awareness can help prevent illness and result in earlier intervention when it does occur. For this reason, health promotion and disease awareness campaigns have great potential to alleviate the global burden of disease. Global Public Health Days (GPHD) are frequently implemented with this intent, but research evaluating their effectiveness, especially in the developing world setting, is scant.

**Objectives:**

We aimed to evaluate the impact of four GPHDs (World Cancer Day, World Diabetes Day, World Mental Health Day, World AIDS Day) on online health information seeking behaviour (OHISB) in five Central and South American (CSA) countries which differ in their stage of economic development and epidemiological transition (Uruguay, Chile, Brazil, Colombia, Nicaragua).

**Methods:**

Google Trends data was used as a ‘surrogate’ of OHISB. This was measured on the 28 days leading up to the GPHD, on the date of the GPHD, and on the seven days following it. The Joinpoint regression programme was used to perform a time trend analysis on the Google Trends data. This allowed us to identify statistically significant time points of a change in trend, which reflect significant ‘changes’ to OHISB.

**Results:**

GPHDs were inconsistently effective at influencing internet search query activity in the studied countries. In situations where an effect was significant, this impact was consistently short-term, with Relative Search Volume level returning to precampaign levels within 7 days of the GPHD.

**Conclusions:**

Our findings imply the need to revise GPHDs or create alternative health awareness campaigns, perhaps with a more long-term approach and tailored to the specific health needs of the CSA population. Developing effective preventive strategies is vital in helping combat the rising threat of NCDs in this region.

Disease prevention and health promotion campaigns are communication activities that aim to raise awareness of specific health topics and influence health behaviour [1]. Global public health days (GPHD) are international health awareness campaigns that aim to draw attention to specific conditions or health-related themes. As a preventive strategy, they have great potential to alleviate the global burden of disease. But despite the frequency with which GPHDs are developed and implemented, relatively little research has examined their reach and impact [2]. The effectiveness of public health initiatives can be difficult to quantify without bespoke, large scale data collection, which is costly, untimely, and often limited in terms of geographical scope [3]. Consequently, alternative real-time health-related surveillance of how people interact with health information disseminated through GPHDs is sought.

As the internet has become a key source for health-related information, online health-information seeking behaviour (OHISB) can be used as a “surrogate” measure of disease awareness in the context of public awareness [4]. Trends in web search terms can provide valuable insight into population health seeking behaviour, as well as collective health trends [5,6]. Through Google Trends (GT; Alphabet Inc, Mountain View CA, USA), a free and publicly accessible tool, it is possible to access such data. GT analyses Google searches, generating data on the geographical and temporal search patterns according to specified keywords [7]. GT determines the proportion of searches for a user specified search term among all searches performed with Google. It uses this data to provide a relative search volume (RSV), which is the query share of a particular term for a given location and time period, normalised by the highest query share of that search term [8].

Most of the developing world is experiencing an epidemiological transition resulting in a triple burden of disease profile. This is the rising prevalence of non-communicable diseases (NCDs), alongside the persisting threat from infectious diseases, as well as accidental injuries and problems related to globalisation [9,10]. Due to significant social, demographic and economic changes that have occurred heterogeneously across Central and South America (CSA), the region is experiencing different stages of the epidemiological transition simultaneously [11,12].

CSA encompasses sociocultural, political and economic diversity, and experiences stark health inequalities despite reforms for universal health coverage [13]. In developed countries, the epidemiological transition is considered to be “complete”; NCDs have prevailed since the 1950s and the threat of infectious diseases is well managed. A more recent epidemiological transition is evident in upper-middle income countries, where the NCD burden picked up in the 1980s. Lower and some upper middle-income countries, are less developed in the epidemiological transition.

A large degree of the disease burden in CSA is attributed to cancer, diabetes mellitus, mental health and HIV/AIDS. They are the target of the GPHDs World Cancer Day, World Diabetes Day, World Mental Health Day, and World AIDS Day. We aim to assess the hypothesized causal relationship between GPHDs and OHISB in five CSA countries (Uruguay, Chile, Brazil, Colombia, Nicaragua) using Google Trends data.

## METHODS

### Google Trends

Google Trends data comes from a sample of the total Google search data, which is categorized, connected to a topic, and anonymised. Searches with special characters, those with a very low search volume, and repeated searches from the same individual over a short period of time are excluded from this data. Each sampled data point is then scaled to the total number of searches done over the selected location and time period. This ‘relative popularity’ is given in the form of a Relative Search Volume (RSV) as a value between 0 and 100 [7]. The RSV data are then presented in a search volume index graph. Google trends data only reflects google searches initiated by the user, not the subsequent online activity in response to the findings of the initial search.

### Documentation of Google Trends use

**Figure 1:**
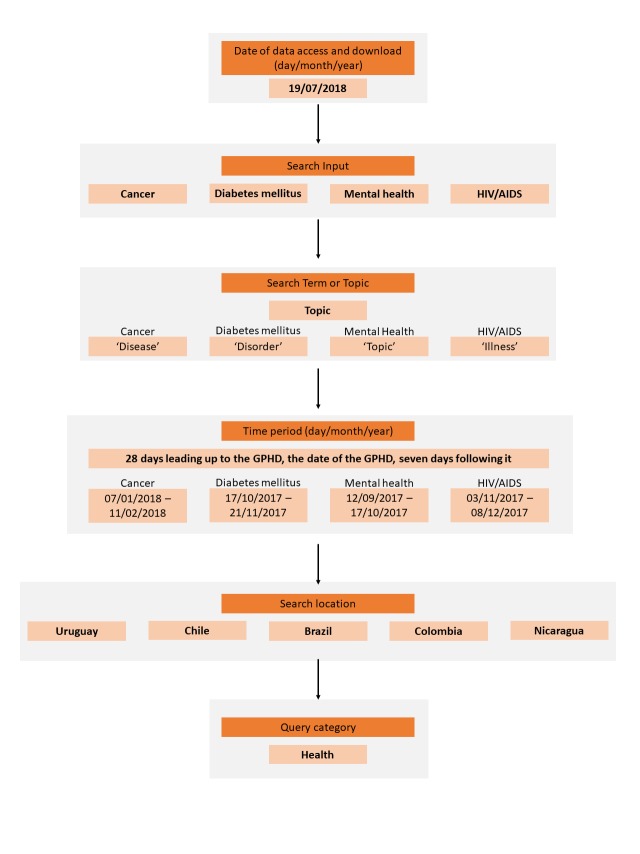
Visual schema of our Google Trends search strategy.

Users can manipulate various aspects of the Google Trends programme to tailor their search. To ensure transparency, reproducibility and quality of our methods, we followed the reporting guidelines recommended by Nuti et al. in 2014 [8]. **Figure 1** is a schema of our search strategy.

### Search input

The full search inputs queried on Google Trends for which data was downloaded were: [Cancer], [Mental health], [Diabetes mellitus], and [HIV/AIDS]. The terms were not used in combination with a plus or minus sign. Search inputs with more than one word were not queried with quotation marks. The use of a capital letter at the start of each search input was consistent across the four searches.

Search inputs were selected to best measure OHISB representative of the GPHD in question. The disease or health concern included in the name of the GPHD was prioritised over a synonym or phrase with a similar meaning. For instance, to investigate OHISB related to Global Mental Health Day, the search term “Mental health” was chosen over alternatives such as “psychological wellbeing”. For World Diabetes Day and World AIDS Day, the search inputs “Diabetes mellitus” and “HIV/AIDS” were selected over “Diabetes” and “AIDS”, as the former search inputs were the ‘topics’ set by Google.

We refined our search by selecting the ‘topic’ instead of the ‘search term’ option. Google describes a topic as “a group of terms that share the same concept in any language”. The example they provide is that searching for “London” as a topic will yield results for searches including “capital of the UK” and “Londres”, the Spanish name for London [14]. The topic feature is thus likely to encompass Google searches querying subtopics or relevant themes of the campaign focus. For instance, our search input “diabetes mellitus” is likely to have included Google Trends data for the search input “gestational diabetes”.

The topic feature encompasses linguistic variations of the search input. This is especially important for our paper as we analysed data from countries with differing official languages; Portuguese in Brazil, and Spanish in Chile, Uruguay, Colombia and Nicaragua. Although ‘diabetes mellitus’ is universal for English, Spanish and Portuguese, the remaining search inputs differ (**Table 1**). Accommodating for linguistic variation also enabled us to measure search inputs in other languages used within studied countries besides the official language. This allows for greater representation of OHISB peri-GPHD.

**Table 1:** Search inputs used for Google Trends searches and their translations into the official languages of the studied countries

Search input (English)	Search input: Spanish	Search input: Portuguese
Cancer	Cáncer	Câncer
Diabetes mellitus	Diabetes mellitus	Diabetes mellitus
Mental health	Salud mental	Saúde mental
HIV/AIDS	VIH/SIDA	VIH/SIDA

### Search variables

Data were accessed and downloaded from Google Trends on July 19th, 2018. The location of the search comprised five CSA countries; two high-income countries (Chile, Uruguay), two upper middle-income countries (Brazil, Colombia), and one lower middle-income country (Nicaragua) [15]. Internet penetrance measured in percentage of the population who are internet users was: Uruguay (66%), Chile (82%), Brazil (61%), Colombia (62%), Nicaragua (25%) [16].

The time scale selected for each Google Trends query was 36 days. This comprised: 28 days leading up to the GPHD, the date of the GPHD, and the seven days following it. Therefore, the exact dates for the time period selected were subject to the calendar date of each awareness day (**Table 2**). The time scale was selected with the intent to capture any effect of campaigns leading up to the GPHD, as well as the duration of any found effect for the 7 days following it.

**Table 2:** Time period and awareness date of each search input

Search input	Time period selected in Google Trends (day/month/year)	Awareness day date (day/month)
Cancer	07/01/2018 – 11/02/2018	04/02
Diabetes mellitus	17/10/2017 – 21/11/2017	14/11
Mental health	12/09/2017 – 17/10/2017	10/10
HIV/AIDS	03/11/2017 – 08/12/2017	01/12

The health query category allowed us to specify the searches within the context of health. Selecting a “query category” instead of using the “search term” option essentially adds another filter to ensure that data represents the intended meaning of the search input. For example, searching for “cancer” under a search term could also include searches that refer to the horoscope instead of the disease.

### Analytic method

A time trend analysis was carried out on the Google Trends data to assess change in RSV as an indicator of OHISB leading up to and following the GPHDs. The joinpoint regression model was used to identify points where a statistically significant change in the linear slope of the trend in the studied time period had occurred. These best-fitting points, called ‘joinpoints’, mark a statistically significant increase or decrease in RSV. Data from the Google Trends Explore page was retrieved in .csv format and opened in Microsoft Excel (Microsoft Inc, Seattle WA, USA). The Joinpoint regression programme was used to undertake the analysis [17]. This is a statistical software that quantitatively identifies time points in which a temporal trend significantly changes, and estimates the regression function with previously identified joinpoints [18]. The analysis was pre-set with the criteria to find a minimum of 0 and maximum of 3 joinpoints. This was to capture an initial increase in RSV when the public health campaign takes place, a second for when trends may down turn after the health campaign, and a third for when the down turn resumes back to the pre-public health campaign RSV. The model selection method was a permutation test, testing for an overall significance level at 0.05. The method applied was a grid search with a minimum of 2 observations from a joinpoint to either end of the data, and between two joinpoints. We did not apply logarithmic transformation of the outcome variable.

## RESULTS

**Figure 2:**
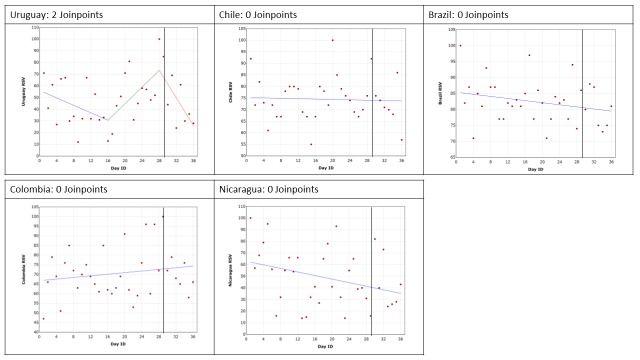
Changes in Relative Search Volume (RSV) leading up to World Cancer Day, World Cancer Day and 7 days following it. Each data point indicates the Relative Search Volume (RSV) measured on the specified day. RSV is the query share of a particular term for a given location and time period, normalised by the highest query share of that search term. Black vertical line marks the day of the Global Public Health Day. Blue = 1^st^ slope, green = 2^nd^ slope, red = 3^rd^ slope, mint green = 4^th^ slope. Number of slopes present depends on the number of joinpoints identified. Joinpoints mark a statistically significant change in the linear slope of the trend in the studied time period.

**Figure 3:**
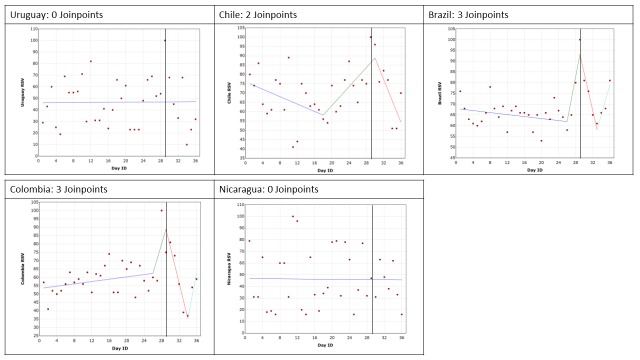
Changes in Relative Search Volume (RSV) leading up to World Diabetes Day, World Diabetes Day and 7 days after it. Each data point indicates the Relative Search Volume (RSV) measured on the specified day. RSV is the query share of a particular term for a given location and time period, normalised by the highest query share of that search term. Black vertical line marks the day of the Global Public Health Day. Colour scheme: Blue = 1^st^ slope, green = 2^nd^ slope, red = 3^rd^ slope, mint green = 4^th^ slope. Number of slopes present depends on the number of joinpoints identified. Joinpoints mark a statistically significant change in the linear slope of the trend in the studied time period.

**Figure 4:**
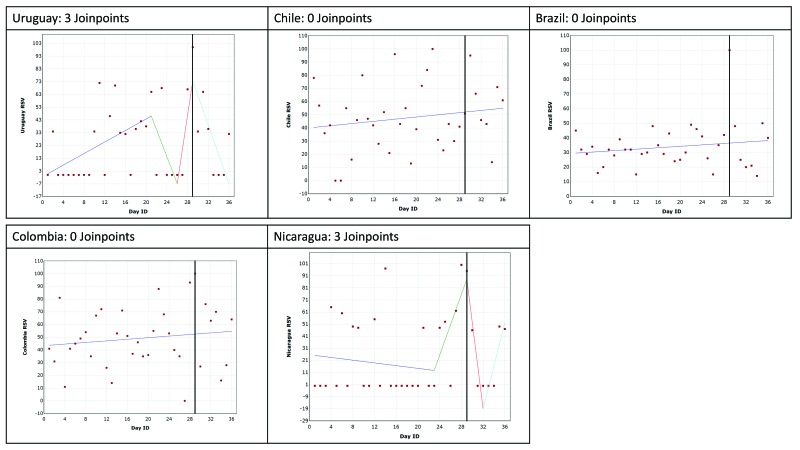
Changes in Relative Search Volume (RSV) leading up to World Mental Health Day, World Mental Health Day and 7 days following it. Each data point indicates the Relative Search Volume (RSV) measured on the specified day. RSV is the query share of a particular term for a given location and time period, normalised by the highest query share of that search term. Black vertical line marks the day of the Global Public Health Day. Colour scheme: Blue = 1^st^ slope, green = 2^nd^ slope, red = 3^rd^ slope, mint green = 4^th^ slope. Number of slopes present depends on the number of joinpoints identified. Joinpoints mark a statistically significant change in the linear slope of the trend in the studied time period.

**Figure 5:**
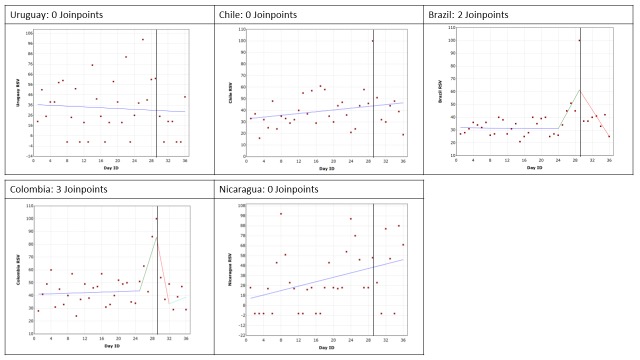
Changes in Relative Search Volume (RSV) leading up to World AIDS Day, World AIDS Day and 7 days after it. Each data point indicates the Relative Search Volume (RSV) measured on the specified day. RSV is the query share of a particular term for a given location and time period, normalised by the highest query share of that search term. Black vertical line marks the day of the Global Public Health Day. Colour scheme: Blue = 1^st^ slope, green = 2^nd^ slope, red = 3^rd^ slope, mint green = 4^th^ slope. Number of slopes present depends on the number of joinpoints identified. Joinpoints mark a statistically significant change in the linear slope of the trend in the studied time period.

Google Trends data are presented for each of the conditions by country. The trend in RSV leading up to, including and following the GPHD for cancer, diabetes mellitus, mental health and HIV/AIDS are shown in **Figure 2**, **Figure 3**, **Figure 4** and **Figure 5**, respectively. The date of the GPHD is represented on the graphs as a black vertical line.

### Cancer and World Cancer Day

The search results are presented in **Figure 2**. In the search results for Uruguay two joinpoints were identified (*P* = 0.01). The first was 13 days before the GPHD, where there was an approximate 43% increase in RSV (*P* = 0.007), reflecting increasing interest leading up to the GPHD. The second joinpoint was 1 day prior to the GPHD, after which there was a decrease in RSV by roughly 47% (*P* = 0.003), representing a decline in attention after the GPHD.

In the analyses for Chile, Brazil, Colombia and Nicaragua no joinpoints were identified. Over the 28 days prior to the GPHD, the day of the GPHD, and the seven days following it, the average RSV value was 74, 82, 70, 49 for Chile, Brazil, Colombia and Uruguay, respectively. However, the data points were very widely spread.

### Diabetes mellitus and World Diabetes Day

The search results are presented in **Figure 3**. The analyses for Brazil and Colombia identified 3 joinpoints (*P* = 0.001 and *P* = 0.0009, respectively), reflecting an increase in searches leading up to the GPHD, a significant decrease following it (Brazil: 35.05%, *P* = 0.03; Colombia: 53.45%, *P* = 0.002), and then an increase back to ‘normal’ or what the RSV may have been prior to the GPHD. This demonstrates that the RSV increase and the OHISB it reflects is relatively short lived.

The results for google searches in Chile found 2 joinpoints (*P* = 0.015), one leading up to the GPHD which marked a significant increase in RSV searches by 31% (*P* = 0.006), and one following the GPHD demonstrating a significant 34.42% decrease in OHISB (*P* = 0.008).

In the analyses for Uruguay and Nicaragua no joinpoints were identified. Over the 28 days prior to the GPHD, the day of the GPHD, and the seven days following it, the average RSV value was 47 and 46 for Uruguay and Nicaragua respectively. However, in both cases the data points were widely spread.

### Mental health and World Mental Health Day

The search results are presented in **Figure 4**. The findings for Uruguay and Nicaragua included 3 joinpoints (*P* = 0.008). Both countries had a joinpoint indicating an increase in RSV searches leading up the GPHD (significant in Uruguay: 45.1%, *P* = 0.009), and then a joinpoint after the GPHD indicating a decrease OHISB (significant in Uruguay: 80.58%, *P* = 0.009). However, there was a large difference between RSV values, with many being 0 and all slopes for Nicaragua and half of those for Uruguay were statistically insignificant.

In the analyses for Chile, Brazil and Colombia no joinpoints were identified. Over the 28 days prior to the GPHD, the day of the GPHD, and the seven days following it, the average RSV value was 48, 34 and 47 for Chile, Brazil and Colombia respectively. However, the data for Colombia and Chile was widely spread.

### HIV/AIDS and World AIDS Day

The search results are presented in **Figure 5**. The findings for Colombia identified 3 joinpoints (*P* = 0.017) reflecting an increase in searches leading up to World AIDS Day, a decrease following it, and then an increase back to “normal” or what the RSV may have been prior to the GPHD. This demonstrates that the RSV increase and the OHISB it reflects is relatively short lived. However, none of the slopes were statistically significant.

The results for google searches in Brazil found 2 joinpoints (*P* = 0.017). One joinpoint marked a significant 30.42% increase in RSV searches leading up the GPHD (*P* = 0.019), and the second joinpoint after the GPHD indicated a significant 36.29% decrease in OHISB (*P* = 0.00001).

In the analyses for Uruguay, Chile and Nicaragua no joinpoints were identified. Over the 28 days prior to the GPHD, the day of the GPHD and the seven days following it, the average RSV value was 33, 40 and 35 for Uruguay, Chile and Nicaragua respectively. However, no slopes were statistically significant and the data for Uruguay and Nicaragua was widely spread.

The results indicate that World AIDS Day only had a significant impact on OHISB in Brazil and Colombia.

## DISCUSSION

The purpose of our study was to assess the hypothesized causal relationship between GPHDs and OHISB in five CSA countries experiencing varying levels of economic development and stages of the epidemiological transition. OHISB was measured with Google trends data. Unlike other authors who have looked at OHISB in response to global public health campaigns [4,19-22], we analysed the google data with the Joinpoint analysis software. This allowed us to identify statistically significant time points of a change in trend, which reflect significant “changes” to OHISB.

### The impact of Global Public Health Days

Our analysis suggests a slight correlation with the disease burden of the campaign’s focus and whether the GPHD had a significant impact on OHISB, but in most cases the findings were inconsistent. The 2018 World Cancer Day was only significant for Uruguay’s OHISB for cancer, which had the greatest proportion of DALYs caused by neoplasms out of all the studied countries. The 2017 World AIDS Day had a significant impact on OHISB in Brazil and Colombia, which had a higher proportion of DALYs caused by HIV than Uruguay and Chile, but not Nicaragua although it had the highest proportion of DALYs caused by HIV out of the studied countries [23].

Surprisingly, despite ‘Diabetes and Chronic Kidney Disease’ being ranked as the top cause of DALYs in Nicaragua, and being included in the top 10 causes of DALYs for all other studied countries [23], the 2017 World Diabetes Day only significantly altered OHISB in Chile, Brazil and Colombia. The findings from the Google Trends data leading up to and following 2017 World Mental Health Day imply a significant impact on OHISB in Uruguay and Nicaragua. ‘Mental disorders’ represented a similar proportion of causes of DALYs in 2017 in the studied countries, and ‘substance use’ to a greater extent in Brazil, Colombia and Nicaragua than in Uruguay and Chile [23].

In cases where GPHDs had a significant impact on OHIBS, this effect was consistently short-term. The relative level of google search queries returned to pre-campaign levels within one week of the campaign. The same effect has been demonstrated in other studies evaluating GPHDs [19,21]. This observation could be due to several reasons. First, the Google Trends data only accounts for a part of OHISB, with the full impact of the campaign manifesting on other online platforms used after Google. Mahabir et al. proposed in 2018 a stimulus-awareness-activism framework, in which one’s obtained awareness leads to online and offline activity related to the concerned topic [24]. In the context of online activity, Mahroum et al assessed in 2018 the digital behaviours in response to a Chikungunya outbreak by analysing the interplay between novel data streams, such as website searches or social networks. GT was found to positively affect twitter activity. Essentially, users tended to search for “Chikungunya” on Google in response to notified cases, and then interacted with the topic on Twitter [25].

Moreover, further awareness can be acquired through browsing key websites related to the topic of the campaign. Users may find these through Google and thus subsequently directly access and use them as a source of health information. For instance, a study assessing the effect of World Blood Donor Day found that as well as increasing the mean RSV for “blood donation”, the blood bank website of the country studied was visited twice as often in the three weeks surrounding the campaign [26]. Considering this, it is likely that the effect of the campaign on OHISB and digital activism lasted longer than our findings show by manifesting in other data streams. Future studies could assess the impact of GPHDs in CSA incorporating more data streams.

### Strengths and limitations

There are limitations with using internet search query data and google trends data specifically as a measure of OHISB. First, only those with internet access can be accounted for in OHISB data. Therefore, our findings are only valid for health information seeking that takes place online. Those without internet access may engage in HISB through alternative means, such as contacting a health professional. However, the percentage of internet users was high in most of the studied countries, only in Nicaragua (25%) the internet penetration was substantially lower compared to that in the other countries and thus could have potentially influenced our results [16]. Despite this, a study on the worldwide Zika-related digital behaviour found that activity came mainly from the CSA region, even though the Zika outbreak breached beyond this region and received global news coverage [27]. Second, the observed interest level is limited to those who use google as a search engine. However, in the studied time period Google represented 97.79% of the search engine market share in South America [28]. These points indicate a sufficient level of internet access and use of Google as search engine in CSA to use Google Trends as a proxy for OHISB.

The over interpretation of trends is discussed as a limitation by Google Trends [7]. Additionally, the calculation of the search value index (Relative Search Volume) is dependent on mathematical assumptions and approximations, which are not public and may obscure true trends in search traffic. However, previous evidence suggests trends have been accurate in approximating the seasonality of conditions [29], and at predicting influenza outbreaks comparable to the US Centres for Disease Control health surveillance mechanisms [30]. A systematic review on the use of Google Trends in health-related research revealed poor documentation of the methodology in most studies, limiting reproducibility of study findings [8]. We have adhered to their documentation recommendations to ensure transparency and reproducibility of our methodology allowing for potential comparisons of findings over time.

### Representativeness of terms for OHSB peri-GPHD

The search input and strategy need to be selected appropriately to yield data that is representative of OHISB in response to GPHDs. Developing a search input to better reflect the studied topic could be achieved by surveying the target population on the search input they would use when googling the theme of interest, as done by Cho et al. in 2013 [31]. However, we sought to measure the OHISB with the actual word(s) used in the campaign name to measure the hypothesized causal relationship between the GPHD and OHISB. Selecting the topic over the search term option allowed us to include data on searches done in different languages and related topics of the search input. However, the Google Trends web page does not reveal what ‘related’ phrases they include in this topic search. Google should make available exactly what search inputs are included for each topic to increase transparency of the search process. This would allow researchers to increase the representation of OHISB peri-GPHD by running separate searches using search inputs that are related but not included in the topic set by Google. Using the query category increases the likelihood of filtering out queries that were not intended for the analysed search input. This option has been chosen and stated in the methods of studies that have used GT to evaluate the impact of World Sepsis Day, the 2015-16 Zika virus outbreak, and economic downturns on searches related to sepsis, Zika, and IVF treatment respectively [4,27,32].

Future studies could improve on our study design to more accurately capture OHISB peri-GPHD by including common misspellings of the search input [33,34]. This would allow for both correctly and incorrectly spelled search inputs to be included in the results. Mavragani 2019 demonstrated that searches using both ‘gonorrhea’ as well as its incorrect spelling ‘gonorrea’ both produced high volumes. However, all possible variations in spelling cannot be included and, in most cases the correct spelling is the most common. Search inputs which carry an accent could be queried without the accent, as this has been shown to make a difference in the search volume results in some languages [35]. In our study, the only search term which carries an accent but is not likely to have been queried without one is “saúde mental”; mental health in Portuguese. Using the cancer search input accounted for searches using the accent free Spanish and Portuguese translation for the word cancer (**Table 1**). Overall, Google should provide more guidance on how to create the optimal strategy with the available search parameters to obtain valid results for a given question.

OHISB and the health awareness obtained, may induce offline health seeking behaviour not measured in our study. Although some evidence has demonstrated an association between online activity and health behaviour [36,37], the relationship cannot be assumed as it is complex and influenced by other variables such as socio-economic status [38]. Health awareness has been shown to trigger behaviour change [39], however, the sustainability of this behaviour is questionable. Public health campaigns, including GPHD, could be used as an opportunity improve uptake of time-limited physical health assessments or interventions with enduring benefits, such as screening or vaccination. World Blood Donor day and World AIDS Day have been shown to significantly increase new donor registration and sales of in-home HIV tests respectively [26,40]. Albeit undoubtedly multifactorial, it has also been demonstrated that behaviours induced by public health campaigns can have a positive impact on clinical outcomes [41-43]. As such, increased knowledge and awareness can be seen as the first step towards health improvement and disease prevention.

### Implications

The aforementioned ways of measuring the effect of GPHDs may reveal a more complete picture of their true impact. However, the inconsistency of their impact on OHISB, and the short-term effect where an effect was significant questions whether GPHDs are effective health awareness campaigns in the CSA region and beyond. A systematic review commissioned by the European Literacy Policy Network identified continuation as a key success factor for awareness raising campaigns [1]. Most themes that GPHDs aim to tackle, such as stigma surrounding mental health or preventive lifestyle changes, comprise cultural and social issues. Addressing them requires long-term strategies [1]. Prolonging the campaign may result in a more sustained effect [20], however activities of the GPHD are organised to take place during the week of the GPHD date, or even during the month. Additionally, one could argue that GPHDs are ‘continuous’ as they take place on an annual basis. The annual reoccurrence of short-term OHISB surges could sum up into a large increase in awareness acquired over years.

The duration of GPHDs varies, and isolated days, weeks or months alone are unlikely to make a substantial change to the campaign’s effectiveness [44,45]. Masiuliene et al. identified in 2015 deliberate and careful targeting as a factor necessary for a successful campaign [1]. This requires defining a gap that needs to be closed, a clear goal and target group, as well as messages customised to engage the targeted audience. GPHD are targeted to some extent, as they choose themes for each year. For instance, the 2018 World AIDS Day theme was ‘Know your status’. However, considering the scale and breadth of populations GPHDs aim to reach, by approaching the specific health needs of population subsets in a culturally appropriate way GPHDs could achieve much greater impact [46]. In Brazil and the USA celebrity disclosure of disease diagnoses have had a larger impact on OHISB and health behaviour than the GPHDs dedicated to the health concern in question [40,47]. Using concrete examples from within the population in question could be the first step towards creating greater and more sustained impact on health awareness.

## CONCLUSIONS

The failure of GPHDs to consistently influence OHISB in a time of high and constantly increasing internet use questions the effectiveness of these awareness campaigns. Our findings suggest more targeted campaigns may be required to improve public awareness and influence health related behaviour. Except for World Cancer Day, impact reports on the studied GPHDs are not published which raises concerns as to whether the campaigns are being evaluated and improved by the coordinating organisations [48]. A transparent revision of the current strategies for GPHDs could be the first step to supporting the continuation of such wide scale and recurring public health campaigns.
